# Platyconic Acid A, a Genuine Triterpenoid Saponin from the Roots of *Platycodon grandiflorum*

**DOI:** 10.3390/molecules13112871

**Published:** 2008-11-18

**Authors:** Yeon Hee Choi, Dae Seok Yoo, Chun whan Choi, Mi-Ran Cha, Young Sup Kim, Hyun Sun Lee, Kang Ro Lee, Shi Yong Ryu

**Affiliations:** 1 Korea Research Institute of Chemical Technology, Taejeon 305-343, Korea. E-Mails: yeonhee@krict.re.kr (Y-H. C.); dsyoo@krict.re.kr (D-S. Y.); choicw@krict.re.kr (C-W. C.); mrcha@krict.re.kr (M-R. C.); yskim@krict.re.kr (Y-S. K.); 2 College of Pharmacy, Sungkyunkwan University, Suwon 440-746, Korea; E-Mail: krlee@skku.ac.kr (K-R. L.); 3 Korea Research Institute of Bioscience & Biotechnology, Taejeon 305-333, Korea; E-mail: leehs@kribb.re.kr (H-S. L.)

**Keywords:** *Platycodon grandiflorum*, Campanulaceae, Platyconic acid A

## Abstract

A genuine triterpenoid saponin, platyconic acid A (**1**) was isolated from the roots extract of *Platycodon grandiflorum*, together with five known saponins: deapioplatycoside E (**2**), platycoside E (**3**), platycodin D_3_ (**4**), platycodin D_2_ (**5**) and platycodin D (**6**). The structure of **1** was determined on the basis of spectral analysis and chemical evidence.

## Introduction

The species *Platycodon grandiflorum* A. DC (Campanulaceae) is a perennial herb found throughout northeast Asia. The roots have been used as food and frequently employed as a folk remedy for bronchitis, asthma, pulmonary tuberculosis, hyperlipidemia, diabetes and inflammatory diseases. Many pharmacological activities of the species such as, cytotoxicity, inhibition of pancreatic lipase, inhibition of nitric oxide synthase and cyclooxygenase II, protection of oxidative hepatotoxicity and cognitive enhancing activity have been reported [1,2,3,4,5,6].

Triterpenoid saponins with unique chemical features on an oleanene backbone were known as the main chemical constituents of the species and more than 30 kinds of saponin components such as platycodin D have been reported so far [7,8,9,10,11,12,13].

In a previous paper, we reported the isolation of a novel triterpenoid saponin, deapioplatycoside E, from the species [1]. In continuation of our phytochemical investigation of the MeOH extract, we have now isolated a new genuine saponin which we have named platyconic acid A (**1**) and identified as platycogenic acid-A 3-*O*-[β-d-glucopyranoside]-28-*O*-[β-d-apiofuranosyl-(1
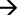
3)-β-d-xylopyranosyl-(1
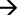
4)-α-l-rhamnopyranosyl-(1
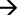
2)-α-l-arabinopyranosyl] ester. It had been reported that two artifacts potentially derived from **1**, a methylester **1a** and a lactonized product, platyconate A lactone (**1b**), were isolated from the extract treated with excess amount of diazomethane [13]. From this result, the authors suggested the presence in the plant extract of the corresponding genuine saponin, **1**, even though they had failed to isolate it. In this paper, we briefly describe an isolation of the genuine

**Figure 1:**
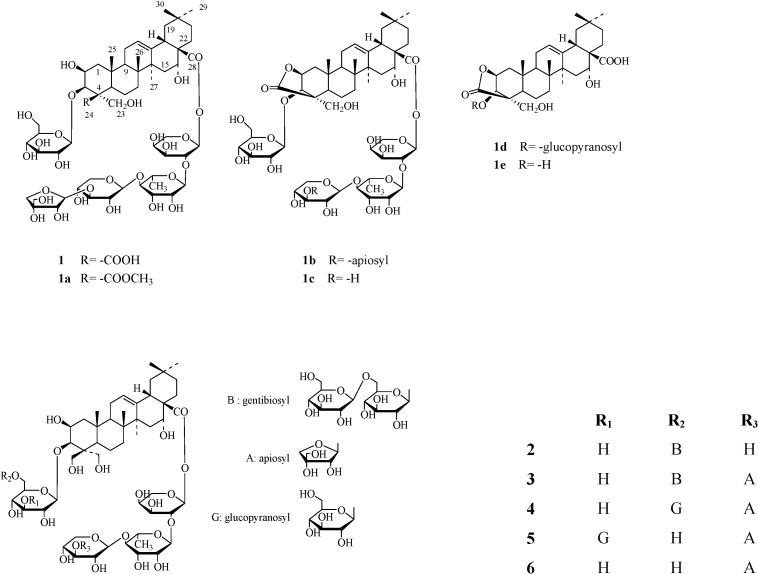
Compounds **1**-**6** from the root extract of *P. grandiflorum*.

saponin, platyconic acid A (**1**) from the roots and its structure elucidation on the basis of spectroscopic analyses and the comparison of ^13^C-NMR data with those of the related saponins **1a-e**, which were obtained by chemical modification of **1** (Figure 1).

## Results and Discussion

The molecular formula of **1** was established as C_57_H_90_O_29_ by MALDI-TOF/MS experiment (*m/z*) 1284 [M+2Na] and ^13^C-NMR data. The IR spectrum showed a hydroxyl group at 3402 cm^-1^ and an ester group at 1726 cm^-1^, respectively. The ^1^H-NMR and ^13^C-NMR spectral data of **1** indicated the presence of a sapogenin 2,3,16,23-tetrahydroxyolean-12-ene-24,28-dioic acid (platycogenic acid A) and oligosaccharide moieties. The ^13^C-NMR spectral data of **1 ** (Table 1) was superimposable with those of the reported platyconic acid A methyl ester (**1a**), the main difference being the methoxy signal (δ 51.3) in **1a**, which was not present in **1**. Instead, the carboxyl signal (δ 175.5) of **1a** was found to be shifted downfield at δ 181.4 (Table 1). These spectral data strongly implied that **1** was an acid congener of **1a**, a genuine saponin. All proton and carbon signals of **1** were assigned by the aid of two-dimensional NMR experiments such as COSY, DEPT, HMQC and HMBC and by the comparison with the data of 1a in the literature [13]. Besides, some chemical modifications of **1** were undertaken to confirm the proposed structure. Compound **1** was found to be easily converted to **1a** by treatment with diazomethane, as mentioned on previous report [13]. On the other hand, the acid hydrolysis of **1** under different conditions afforded two kinds of prosapogenin, **1c**, **1d**, and an artificial sapogenin **1e** (platycogenic acid-A lactone), all of which provided evidence supporting the proposed structure of **1** [8]. From these results obtained from the spectroscopic data and chemical evidence, the structure of **1**

**Table 1:** ^13^C-NMR spectroscopic data (δ) of **1**-**1e**.

	1	1a	1b	1c	1d	1e
C-1	46.7	45.8	41.6	41.7	41.4	42.0
C-2	69.6	69.8	82.8	83.6	83.5	84.6
C-3	83.3	84.4	89.8	89.5	89.5	81.8
C-4	56.3	56.1	53.9	54.5	54.5	55.2
C-5	49.6	50.1	52.5	52.3	52.2	52.1
C-6	20.3	20.5	19.5	19.8	19.7	20.1
C-7	33.7	33.7	33.6	33.9	33.8	34.0
C-8	40.0	40.3	40.7	40.8	40.6	38.2
C-9	47.2	47.6	48.5	48.5	48.5	49.1
C-10	37.2	37.4	37.9	38.0	38.0	36.9
C-11	24.4	24.4	24.7	25.0	25.0	25.2
C-12	122.9	123.0	122.3	122.6	122.2	121.3
C-13	144.2	144.4	145.0	145.6	146.2	147.3
C-14	42.2	42.4	42.5	42.7	42.7	43.1
C-15	36.2	36.1	36.0	36.5	36.9	36.7
C-16	73.8	74.1	73.9	74.4	75.0	75.9
C-17	49.5	50.1	50.0	50.2	49.3	50.8
C-18	41.3	41.6	41.6	41.6	41.9	40.9
C-19	47.1	47.2	47.2	47.6	47.7	48.1
C-20	30.6	30.8	30.7	30.8	30.3	28.3
C-21	35.9	36.1	36.0	36.4	36.8	34.2
C-22	31.7	31.4	31.3	31.4	31.5	31.5
C-23	63.5	64.5	57.5	57.6	57.5	58.3
C-24	181.4	175.5	177.7	178.6	178.6	179.5
C-25	16.1	15.8	17.4	17.8	17.7	18.3
C-26	17.4	17.6	18.1	18.5	18.4	19.1
C-27	27.0	27.2	27.4	27.7	27.7	26.9
C-28	175.8	175.8	175.8	176.3	180.4	185.0
C-29	33.3	33.1	33.0	33.7	33.3	31.7
C-30	24.8	25.2	25.2	25.2	25.2	25.7
24-OCH_3_		51.3				
	**1**	**1a**	**1b**	**1c**	**1d**	
glucose						
C-1	106.0	106.3	105.1	105.7	105.7	
C-2	74.8	75.3	75.0	75.6	75.7	
C-3	78.2	78.5	78.2	79.0	79.2	
C-4	71.8	72.0	71.8	71.5	71.8	
C-5	78.2	78.1	78.2	78.8	78.8	
C-6	61.8	63.0	62.9	63.0	63.0	
arabinose						
C-1	93.4	93.7	93.7	94.1		
C-2	75.5	75.7	75.7	75.8		
C-3	70.3	70.1	70.2	70.8		
C-4	66.2	65.8	65.9	66.8		
C-5	61.8	63.0	62.9	63.8		
rhamnose						
C-1	101.2	101.1	101.1	101.7		
C-2	71.8	72.0	72.0	72.4		
C-3	72.3	72.4	72.4	73.2		
C-4	83.3	83.6	83.6	84.3		
C-5	68.4	68.7	68.6	69.1		
C-6	18.4	18.1	18.3	18.8		
xylose						
C-1	106.2	106.5	106.5	107.4		
C-2	74.7	75.0	75.0	76.2		
C-3	85.2	85.5	85.6	79.2		
C-4	68.9	69.5	69.5	71.8	
C-5	66.5	66.8	66.8	67.9	
apiose					
C-1	110.8	111.2	111.2		
C-2	77.8	77.9	77.9		
C-3	80.1	80.0	80.0		
C-4	74.3	75.0	75.0		
C-5	65.1	65.8	65.7		

Data for **1a** and **1b** from ref. [13]

was determined unambiguously as a that of the genuine saponin, platyconic acid A (platycogenic acid-A 3-*O*-β-d-glucopyranoside]-28-*O*-[β-d-apiofuranosyl-(1
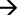
3)-β-d-xylopyranosyl-(1
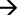
4)-α-l-rhamno- pyranosyl-(1
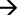
2)-α-l-arabinopyranoside. This is the first report on the isolation of compound **1** from natural sources.

**Figure 2:**
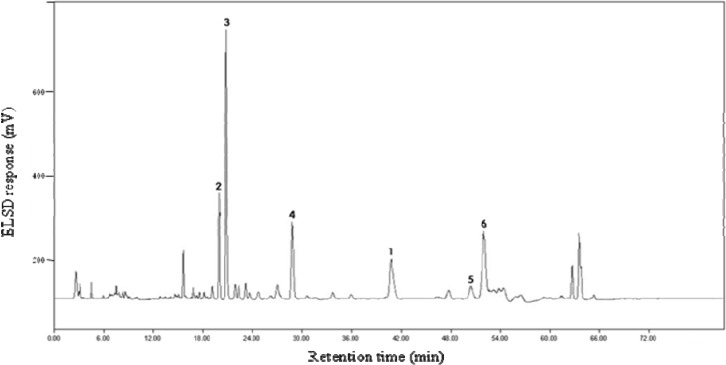
HPLC profile of saponin fraction from the MeOH root extract of *P. grandiflorum*.

Figure 2 shows the HPLC profile of the MeOH root extract.

Platyconic acid A (**1**), deapioplatycoside E (**2**), platycoside E (**3**), platycodin D_3_ (**4**), platycodin D_2_ (**5**) and platycodin D (**6**).

## Conclusions

During the phytochemical survey of the roots extract of *Platycodon grandiflorum*, a new triterpenoid saponin **1** was isolated and identified on the basis of spectral analysis and chemical evidence as a genuine saponin, which we have named platyconic acid A.

## Experimental

### General

Optical rotations were determined using a Rudolph Autopol IV polarimeter. MS spectra were measured on a Voyager PE Biosystems USA MALDI-TOF/MS Spectrometer. ^1^H-NMR and ^13^C-NMR spectra was recorded on a Brucker AVANCE 800 NMR spectrometer using TMS as an internal standard. Preparative HPLC used a Futecs NS-3000i system equipped with an Optimapak column (50 x 250 mm, 10 μm, RSTech) and a ELSD (sofTA, USA). 

### Plant Material

The species, *P. grandiflorum* cultivated for three years at the mountainside of Kyungnam Province, Korea was harvested in September 2003. A voucher specimen (Herbarium No. JS-03024) has been preserved at the Herbarium of JangSaeng Doraji Co. LTD., Jinju, Korea.

### Extraction and Isolation

Dried roots of *P. grandiflorum* (5 kg) were extracted three times with methanol at room temperature for 7 days. Concentration of the solvent gave a brown syrupy extract (1.4 kg) which was suspended in water and then partitioned successively with ethyl acetate (63 g) and *n*-butanol (130 g). The *n*-butanol layer was suspended in H_2_O (2 L) and poured onto a Diaion HP-20 column (Φ = 5.0 × 100 cm), which was stabilized with H_2_O. The column was washed with H_2_O (2 L) and then eluted with MeOH (5 L). The eluate was concentrated in a reduced pressure to give a crude saponin mixture (75 g).

### Characterization of platyconic acid A (**1**)

Obtained as a white amorphous powder, 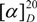 = - 8.89 (c 1, MeOH); IR ν_max_: 3402, 2927, 1726, 1076 and 1033 cm^-1^; MALDI-TOF/MS *m/z*: 1284 [M+2Na]; C_57_H_90_O_29_; ^1^H-NMR (pyridine-*d_5_*, 800 MHz): δ 0.98, 1.10, 1.52, 1.69, 1.75 (each 3H, H-25, 26, 27, 29, 30), 2.75 (1H, d, *J* = 13.3Hz, H-18), 5.61 (1H, brs, H-12); ^13^C-NMR (pyridine-*d_5_*, 200 MHz): see Table 1. 

### Preparation of **1a** by treatment of **1** with diazomethane

Compound **1** (200 mg) was dissolved in MeOH (10 mL), to which 10 equivalents of CH_2_N_2_ in ether was added and the resulting mixture was stirred overnight, then it was evaporated to dryness and purified by ODS gel column chromatography (MeOH-H_2_O) to afford **1a** (140 mg) as an amorphous powder.

### Preparation of **1c** and **1d** by partial hydrolysis of **1**

Compound **1** (100 mg) was dissolved in 0.1 N HCl in MeOH (10 mL) and stirred at room temperature overnight. The solvent was removed under nitrogen to provide a colorless solid, which was purified by ODS to afford **1c** (60 mg) as an amorphous powder. In a similar manner, **1** (100 mg) was dissolved in 1 N HCl in MeOH (10 mL) and refluxed on water bath for 1 hr. The reaction mixture was concentrated *in vacuo* to yield a colorless solid which was purified by ODS column chromatography to afford **1d** (42 mg) as an amorphous powder.

### Preparation of **1e** by acid hydrolysis of **1**

Compound **1** (100 mg) was dissolved in 4 N HCl in MeOH (10 mL) and refluxed on a water bath for 6 hr. The reaction mixture was concentrated *in vacuo* to yield a colorless solid which was purified by ODS column chromatography to afford **1e** (21 mg) as an amorphous powder.

### HPLC analysis of the roots extract of P. grandiflorum

A portion of the saponin fraction prepared from the roots extract of *P. grandiflorum* as described above in *Extraction and Isolation* (20 g) was purified by repeated preparative HPLC. The residue was dissolved in distilled water and passed through a solid-phase-extraction cartridge (RP-C_18_, CEREX No. 600-3506). The cartridge was washed with excess amount of distilled water and eluted with MeOH. The MeOH solution was injected onto an Optimapak column (4.6 x 250 mm, 5 µm, RSTech) maintained at 40°C on a Futecs NS-3000i system equipped with an ELSD detector (sofTA, USA). A mixture of 50 mM ammonium acetate solution (NH_4_Ac), acetonitrile and methanol was used as mobile phase as follows: eluent A = 85:10:5 NH_4_Ac-acetonitrile-methanol; eluent B = 55:40:5 NH_4_Ac-acetonitrile-methanol; flow rate: 0.8 mL/min; gradient: 0-5 min (0-15% B), 5-28 min (15-38% B), 28-33 min (38-40% B), 33-53 min (40-43% B), 53-63 min (43-60% B), 63-81 min (60-100% B). This allowed isolation of the six triterpenoid saponins **1**-**6**, *i.e.*, 660 mg of **1** (t_R_ 42.8 min), 420 mg of **2** (t_R_ 19.1 min), 1,450 mg of **3** (t_R_ 20.3 min), 320 mg of **4** (t_R_ 28.4 min), 56 mg of **5** (t_R_ 49.5 min) and 980 mg of **6** (t_R_ 51.4 min) (Figure 2). Compounds **2**-**6** were identified by direct comparison of their physical and spectral data (^1^H-NMR and ^13^C-NMR) with those in the literature [1,13,14,15].

## References

[1] Kim Y.S., Kim J.S., Choi S.U., Kim J.S., Lee H.S., Roh S.H., Jeong Y.C., Kim Y.K., Ryu S.Y. (2005). Isolation of a new saponin and cytotoxic effect of saponins from the root of *Platycodon grandiflorum* on human tumor cell lines. Planta Med..

[2] Xu B.J., Han L.K., Zheng Y.N., Lee J.H., Sung C.K. (2005). In vitro inhibitory effect of triterpenoidal saponins from Platycodi Radix on pancreatic lipase. Arch. Pharm. Res..

[3] Ahn K.S., Noh E.J., Zhao H.L., Jung S.H., Kang S.S., Kim Y.S. (2005). Inhibition of inducible nitric oxide synthase and cyclooxygenase II by *Platycodon grandiflorum* saponins via suppression of nuclear factor-kappaB activation in RAW 264.7 cells. Life Sci..

[4] Lee K.J., Choi C.Y., Chung Y.C., Kim Y.S., Ryu S.Y., Roh S.H., Jeong H.G. (2004). Protective effect of saponins derived from roots of *Platycodon grandiflorum* on tert-butyl hydroperoxide-induced oxidative hepatotoxicity. Toxicol. Lett..

[5] Kim Y.S., Kang J.S., Kim J.S., Choi Y.H., Seo J.H., Lee J.W., Kim S.K., Lee H.S., Cho Y.S., Roh S.H., Jeong Y.C., Shim K.H., Ryu S.Y. (2004). Ameliorating effect of the root extract from *Platycodon grandiflorum* on the ethanol-induced cognitive impairment in mice. Kor. J. Pharmacogn..

[6] Choi Y.H., Kim Y.S., Yeo S.J., Roh S.H., Jeong Y.C., Kang J.S., Ryu S.Y. (2008). Ameliorating effect of balloon flower saponin on the ethanol-induced memory impairment in mice. Phytotherapy. Res..

[7] He Z., Qiao C., Han Q., Wang Y., Ye W., Xu H. (2005). New triterpenoid saponins from the roots of *Platycodon grandiflorum*. Tetrahedron.

[8] Fu W.W., Shimizu N., Takeda T., Dou D.Q., Chen B, Pei Y.H., Chen Y.J. (2006). New A-ring lactone triterpenoid saponins from the roots of *Platycodon grandiflorum*. Chem. Pharm. Bull..

[9] Fu W.W., Shimizu N., Dou D.Q., Takeda T., Fu R., Pei Y.H., Chen Y.J. (2006). Five new triterpenoid saponins from the roots of *Platycodon grandiflorum*. Chem. Pharm. Bull..

[10] Ishii H., Tori K., Tozyo T., Yoshimura Y. (1978). Structures of polygalacin-D and -D_2_, platycodin-D and -D_2_, and their monoacetates, saponins isolated from *Platycodon grandiflorum* A. DC. determined by carbon-13 nuclear magnetic resonance spectroscopy. Chem. Pharm. Bull..

[11] Fu W.W., Dou D.Q., Shimizu N., Takeda T., Pei Y.H., Chen Y.J. (2006). Studies on the chemical constituents from the roots of *Platycodon grandiflorum*. J. Nat. Med..

[12] Li W., Xiang L., Zhang J., Zheng Y.N., Han L.K., Saoto M. (2007). A new triterpenoid saponin from the roots of *Platycodon grandiflorum*. Chin. Chem. Lett..

[13] Ishii H., Tori K., Tozyo T., Yoshimura Y. (1984). Saponins from roots of *Platycodon grandiflorum*. Part 2. isolation and structure of new triterpene glycosides. J. Chem. Soc. Perkin. Trans. I.

[14] Nikaido T., Koike K., Mitsunaga K., Saeki T. (1999). Two new triterpenoid saponins from *Platycodon grandiflorum*. Chem. Pharm. Bull..

[15] Ishii H., Tori K., Tozyo T., Yoshimura Y. (1981). Saponins from roots of *Platycodon grandiflorum*. part 1. structure of prosapogenin. J. Chem. Soc. Perkin. Trans. I.

